# Characterization of the complete chloroplast genome of medicinal tea tree (*Melaleuca alternifolia*)

**DOI:** 10.1080/23802359.2019.1673246

**Published:** 2019-10-01

**Authors:** Hailong Liu, Maolin Geng, Yufeng Qin, Yufei Xiao, Mimi Li

**Affiliations:** a Guangxi Key Laboratory of Superior Timber Trees Resource Cultivation, Guangxi Forestry Research Institute, Nanning, China;; b Institute of Botany, Jiangsu Province and Chinese Academy of Sciences, Nanjing, China;; c The Jiangsu Provincial Platform for Conservation and Utilization of Agricultural Germplasm, Nanjing, China

**Keywords:** *Melaleuca alternifolia*, Myrtaceae, chloroplast genome, medicinal tea tree

## Abstract

*Melaleuca alternifolia* is commonly known as the medicinal tea tree. The complete chloroplast (cp) genome sequence is 160,104 bp in length, with a quantitative molecule structure comprising two copies of inverted repeats (IRa and IRb) of 26,737 bp separated by a large single copy (LSC) of 88,151bp, a small single copy (SSC) of 18,479 bp. A total of 131 genes were identified including 84 protein-coding genes, 37 tRNA genes, eight rRNA genes and two pseudogene (Ψ*ycf*1, Ψ*inf*A), respectively. Phylogenomic analysis suggests that *M. alternifolia* is closely related to the rest species of Myrtaceae with strong bootstrap values.

*Melaleuca alternifolia* (Maiden & Betche) Cheel (Myrtaceae), commonly known as medicinal tea tree, is a commercially important tall shrub native to eastern Australia that produces valuable tea oil (Butcher et al. 1995). However, *M. dissitiflora*, *M. linariifolia*, and other species of *Melaleuca* can also be used to obtain foliar essential oil (Sharifi-Rad et al. 2017). Correct identification of *Melaleuca* is a prerequisite because only terpinen-4-ol chemotype of *M. alternifolia* yields a major medicinally and industry values (Carson et al. 2006). Chloroplast (cp) genome sequences can harbour useful molecular information for species identification and valuable gene sources for the breeding of *M. alternifolia*.

Fresh leaves were collected from Guangxi Forestry Research Institute (22°55′32.89″N, 108°21′2.20″E) and the voucher specimen (accession No.: 617001) was deposited in the Herbarium of Institute of Botany, Jiangsu Province and Chinese Academy of Sciences (NAS). The total genomic DNA was isolated using modified CTAB method (Doyle and Doyle 1987) and sequenced on Illumina HiseqXten platform (San Diego, CA). The pair-end sequencing data was assembled using NOVOPlasty 2.7.2 (Dierckxsens et al. 2017). Annotation of the *M. alternifolia* cp genome was performed using GeSeq (https://chlorobox.mpimp-golm.mpg.de/geseq.html) with default settings and manually adjusted coding regions in Geneious 11.1.5 (https://www.geneious.com).

The cp genome sequence of *M. alternifolia* (Genbank accession number: MN310606) was 160,104 bp in length. Two copies of inverted repeat (IRs, 26,737 bp) separated the rest of cp genome sequence into a large single copy (LSC, 88,151bp) and a small single copy (SSC, 18,479 bp). The overall GC content of *M. alternifolia* cp genome was 36.7%. And the base composition of the cp genome is A (31.3%), T (32.0%), C (18.7%), and G (18.0%). A total of 131 genes were identified, containing 84 protein-coding genes, 37 transfer RNA genes (tRNA), eight ribosomal RNA genes (rRNA) and two pseudogene (Ψ*ycf*1, Ψ*inf*A). Among these genes, 13 protein-coding genes contained one or two introns.

In order to test the phylogenomic relationship of *M. alternifolia* with other Myrtaceae, the whole cp genomes of previously published species of Myrtaceae and one outgroup (*Melastoma candidum*, Melastomataceae, KY745894) were aligned using the MAFFT 7.409 (Katoh and Standley 2013). And molecular phylogenetic tree were reconstructed with the maximum likelihood method that employed in RAxML (Stamatakis 2014). Phylogenomic analysis suggests that *M. alternifolia* is closely related to the rest species of Myrtaceae with strong bootstrap values (Figure 1).

**Figure 1.**
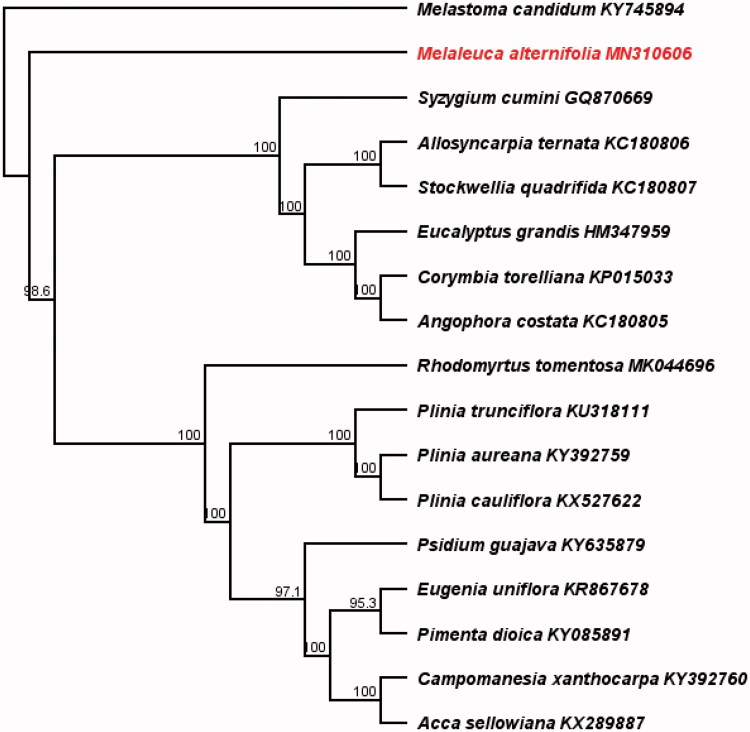
The consensus RAxML bootstrapping tree of *Melaleuca alternifolia* and other Myrtaceae species based on the complete chloroplast genomes. The number above each node indicated the bootstrap support value.
